# Ozone augments interleukin-8 production induced by ambient particulate matter

**DOI:** 10.1186/s41021-018-0102-7

**Published:** 2018-07-18

**Authors:** Jun Kurai, Kunishige Onuma, Hiroyuki Sano, Futoshi Okada, Masanari Watanabe

**Affiliations:** 1 Department of Respiratory Medicine and Rheumatology, Tottori University Hospital, 36-1 Nishichou, Yonago, Tottori, 683-8504 Japan; 2 Division of Pathological Biochemistry, Department of Biomedical Sciences, Faculty of Medicine, Tottori University, 86 Nishi-cho, Yonago, Tottori, 683-8503 Japan; 3 Department of Respiratory Medicine and Allergology, Kinki University Faculty of Medicine, 377-2 Ohnohigashi, Osakasayama, Osaka, 589-0014 Japan

**Keywords:** Antioxidant, Interleukin-6, Interleukin-8, Ozone, Particulate matter, Pro-inflammatory cytokine, Reactive oxygen species

## Abstract

**Background:**

Experimental and controlled human exposure studies have demonstrated additive effects of ambient particulate matter and ozone on health. A few epidemiological studies have suggested that ambient particulate matter components are important for the combined effects of ambient particulate matter and ozone on health. However, few studies have examined whether ozone changes the effects of ambient particulate matter on pro-inflammatory cytokine production. In this study, the influence of ozone on pro-inflammatory cytokine production in response to ambient particulate matter was evaluated.

**Results:**

Ambient particulate matter smaller than 1 μm was collected and the suspension of this particulate matter was bubbled through 0.12 ppm and 0.24 ppm ozone. THP1 cells were stimulated by the solution containing the particulate matter with and without bubbling through ozone at 1 μg/mL. The interleukin-8 concentrations in the supernatants of THP1 cells stimulated by collected particulate matter dissolved in solution were 108.3 ± 24.7 pg/mL without ozone exposure, 165.0 ± 26.1 pg/mL for 0.12 ppm ozone bubbling for 1 min, 175.1 ± 33.1 pg/mL for 0.12 ppm for 5 min, 183.3 ± 17.8 pg/mL for 0.12 ppm for 15 min, 167.8 ± 35.9 pg/mL for 0.24 ppm for 1 min, 209.2 ± 8.4 pg/mL for 0.24 ppm for 5 min, and 209.3 ± 14.3 pg/mL for 0.24 ppm for 15 min. Ozone significantly increased interleukin-8 concentrations compared to those for particulate matter dissolved in solution without ozone exposure and the solvent only (8.2 ± 0.9 pg/mL) in an ozone concentration-dependent manner. Collected particulate matter in solutions with or without bubbling through ozone had no effect on interleukin-6 production. The antioxidant *N*-acetyl-_L_-cysteine significantly inhibited the increases in interleukin-8 induced by solutions with particulate matter, regardless of ozone exposure. The reactive oxygen species concentration in solutions with collected particulate matter was not associated with ozone bubbling.

**Conclusion:**

Ozone may augment the production of interleukin-8 in response to ambient particulate matter by a mechanism unrelated to reactive oxygen species. These results support the epidemiological evidence for combined effects of ambient particulate matter and ozone on human health.

## Background

Ambient particulate matter (PM) and ozone are among the six most common air pollutants. Among these pollutants, ambient PM, especially fine PM, with a median aerodynamic diameter of less than 2.5 μm, has the greatest effect on human health. Numerous epidemiological studies have indicated that exposure to ambient PM is correlated with increased respiratory and cardiovascular morbidity and mortality [1–4]. Similarly, short-term exposure to ambient PM is associated with hospital admission, respiratory function, and respiratory symptoms in patients with respiratory and cardiovascular diseases [4–6]. There is far less evidence for the effect of ozone on health than for an effect of PM, but numerous studies have suggested that breathing ground-level ozone has harmful effects [7–11].

The toxicological mechanisms underlying the effects of PM and ozone on human health have gradually been clarified. There is extensive evidence for associations between PM and intermediary inflammatory outcomes [12–14]. According to these in vitro and in vivo analyses, after PM is engulfed or phagocytized by cells, various pro-inflammatory mediators, such as interleukin (IL)-6 and IL-8, are secreted by cells [12–14]. Similarly, numerous studies have found increases in the concentrations of various pro-inflammatory mediators, such as interleukin IL-6 and IL-8, in humans exposed to ozone [15–18]. The generation of these pro-inflammatory mediators may be explained by reactions with the airway epithelial lining fluid and cell membranes [19–21].

Multiple air pollutants are continually generated; however, ozone and PM are traditionally considered separate problems and both epidemiological and experimental studies have mainly focused the adverse health effects of single air pollutants. Recent experimental studies have shown combined effects of fine PM and ozone on health [15, 22–26]. Several controlled human exposure studies have also demonstrated additive effects of fine PM and ozone on human health [12, 27–30]. A few epidemiological studies have suggested that fine PM components are important for these combined effects [31–33] because the chemical composition of PM varies geographically and seasonally [34–37].

Previous experimental and controlled human exposure studies have typically evaluated the separate administration of ozone and ambient PM to cells, animals, and humans. Ozone and ambient PM are chemically coupled in the ambient air [38] and ozone may affect inflammation induced by ambient PM. In the present study, whether ozone influences the effects of PM on pro-inflammatory cytokine production was examined by stimulating cells with PM that was first exposed to ozone.

## Methods

### Preparation of airborne particles and ozone exposure

From March 12 to March 20, 2018, airborne PM smaller than 1.1 μm was collected on a 20 × 25 cm quartz filter (Poreflon; Sumitomo Electric, Osaka, Japan) at a flow rate of 566 L/min using a high-volume air sampler with a diverter (high-volume air sampler, Andersen type; Shibata, Saitama, Japan) for 72 h. To extract collected PM, these filters were extracted at 10 μg/mL PM with distilled deionized water using an ultrasonic apparatus (BRANSONIC2800; Emerson Japan, Atsugi, Japan) for 60 min [39, 40]. The extraction liquids were filtered through 1-μm filters (pluriSelect, Leipzig, Germany). PM smaller than 1 μm in diameter dissolved in solution was blended from March 12 to March 20, 2018 and sterilized at 121 °C for 30 min in an autoclave (Tomy SX-300; Tomy, Tokyo, Japan). The extraction liquids of collected PM were stored in a freezer at − 70 °C to prevent the growth of bacteria and fungi.

The extraction liquids of collected PM and distilled deionized water were bubbled through 0.12 ppm or 0.24 ppm ozone at 5 L/min using an air flow controller (RAPIFLOW; CKD, Aichi, Japan) for 1 min, 5 min, and 15 min. Using an ozonizer (OHNIT, Okayama, Japan), ozone was generated in 0.11 and 0.22 s at intervals of 7 s and adjusted to 0.12 ppm and 0.24 ppm using the air flow controller. The pH of the extraction liquids of collected PM and distilled deionized water was measured before and after bubbling through ozone.

### Particulate matter exposure and the measurement of Il-6 and IL–8 production

In vitro toxicological studies and methods to measure cytokine production induced by PM using airway epithelial cells and monocytes are well established [41]. THP1, a human monocyte cell line, was used to measure cytokine production induced by collected fine PM. These cells were cultured in Roswell Park Memorial Institute medium 1640 containing 10% fetal bovine serum, 0.05 mM 2-mercaptoethanol, 100 U/mL penicillin, 100 μg/mL streptomycin, and 0.5 μg/mL amphotericin B at 37 °C and 5% CO_2_ and in a humidified cell culture incubator.

THP1 cells (1 × 10^5^ cells/270 μL/well) in a 96-well polystyrene plate (Sumitomo Bakelite, Tokyo, Japan) were stimulated for 24 h at 37 °C with 30 μL of solvent only (negative control), 30 μL of distilled deionized water, 30 μL of distilled deionized water after bubbling through ozone, 30 μL of 10 μg/mL collected PM in solution, and 30 μL of 10 μg/mL collected PM dissolved in solution after bubbled through ozone. For distilled deionized water and collected PM dissolved in solution after bubbling through 0.24 ppm ozone, THP1 cells were pretreated with 5 mM and 25 mM *N*-acetyl-_L_-cysteine (NAC) for 30 min at 37 °C to confirm the generation of reactive oxygen species (ROS). After the administration of distilled deionized water and collected PM dissolved in solution with and without bubbling through ozone, the pH of each supernatant was measured. After 24 h of exposure, the viability of THP1 cells exceeded 95% for all samples, as assessed using a trypan blue-exclusion test. After 24 h of exposure, supernatants were removed and centrifuged at 250×*g* to remove floating cells and then at 2500×*g* to remove the remaining particles. The final supernatants were stored at − 70 °C.

The IL-6 and IL-8 concentrations in the supernatants were measured using enzyme-linked immunosorbent assay (ELISA) kits for IL-6 and IL-8 (R&D Systems, Minneapolis, MN, USA), according to the manufacturer’s protocols, with 96-well plates (Seikagaku, Tokyo, Japan). Samples were run in duplicate and read using an automated ELISA reader (Model 680; Bio-Rad, Hercules, CA, USA).

### Detection of reactive oxygen species

The generation of ROS in distilled deionized water and collected PM dissolved in solution with and without bubbling through ozone was determined using 2′,7′-dichlorodihydrofluorescein diacetate (H_2_DCFDA) reagent (D-399; Molecular Probes, Tokyo, Japan) [41]. An equal amount of Roswell Park Memorial Institute medium containing 10 mol/L dichlorodihydrofluorescein, which was converted from H_2_DCFDA reagent by treatment with 0.2 mol/L sodium hydroxide, was added to 100 μL of distilled deionized water and collected PM dissolved in solution with and without bubbling through ozone. An oxidized fluorescent product, dichlorofluorescein, was detected using a fluorescence reader, Infinite M 200 Pro (TECAN Japan, Kawasaki, Japan), at an excitation wavelength of 504 nm and an emission wavelength of 530 nm, and monitored every 5 min.

### Statistical analysis

Four independent experiments were performed for each analysis and a one-way or two-way ANOVA was used to evaluate statistical significance. *P* < 0.05 was considered statistically significant.

## Results

### Production of IL-6 and IL-8

**Fig. 1:**
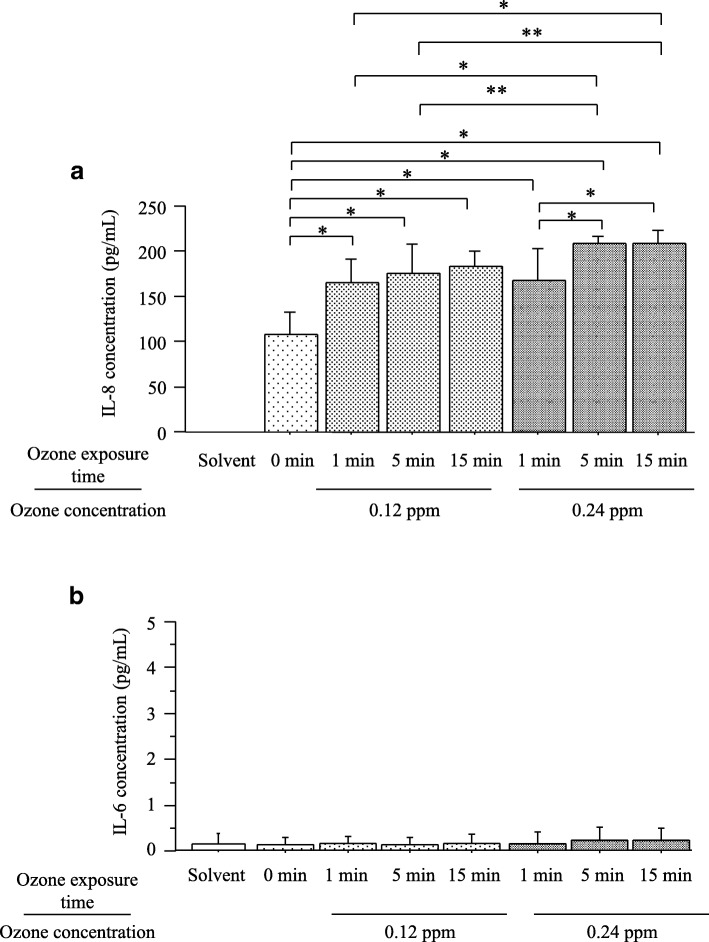
Concentrations of interleukin (IL)-8 (**a**) and IL-6 (**b**) in the supernatants of THP1 cells stimulated by collected particulate matter (PM) dissolved in solution (1 μg/mL) with and without bubbling through ozone. Bars represent means ± SD. *P < 0.01 and **P < 0.05

The pH levels of the collected PM dissolved in solution with and without bubbling through ozone ranged from 3.0 to 3.3. When 30 μL of collected PM dissolved in solution was added to 270 μL of the THP1 cell suspension, the pH of the cell culture medium ranged from 7.1 to 7.4. The cell viability exceeded 95% after 24 h of stimulation by all collected PM dissolved in solution with and without bubbling through ozone. Allowing for the simultaneous quantitative measurement of IL-6 and IL-8, the exposure of THP1 cells to collected PM dissolved in solution (1 μg/mL) with and without bubbling through ozone significantly induced the production of IL-8, but not IL-6 (Fig. 1). The concentrations of IL-8 in the supernatants of THP1 cells stimulated by collected PM dissolved in solution were 108.3 ± 24.7 pg/mL for PM without ozone exposure, 165.0 ± 26.1 pg/mL for 0.12 ppm ozone bubbling for 1 min, 175.1 ± 33.1 pg/mL for 0.12 ppm for 5 min, 183.3 ± 17.8 pg/mL for 0.12 ppm for 15 min, 167.8 ± 35.9 pg/mL for 0.24 ppm for 1 min, 209.2 ± 8.4 pg/mL for 0.24 ppm for 5 min, and 209.3 ± 14.3 pg/mL for 0.24 ppm for 15 min. There were significant differences in the increase in IL-8 caused by all collected PM dissolved in solution with and without bubbling through ozone compared with concentrations induced by the solvent only (distilled deionized water, 8.2 ± 0.9 pg/mL) (Fig. 1a). In contrast, both collected PM dissolved in solution with and without bubbling through ozone did not increase the concentration of IL-6 compared with that for the solvent only (Fig. 1b). The observed concentrations of IL-8 in collected PM dissolved in solutions with bubbling ozone were significantly higher than those without bubbling through ozone. There were significant differences in the increase in IL-8 by collected PM dissolved in solutions for 5 min and 15 min with bubbling through 0.24 ppm ozone compared with those for solutions bubbled through 0.12 ppm ozone for 1 min and 5 min. The concentrations of IL-8 produced by collected PM dissolved in solutions for 5 min and 15 min with bubbling through 0.24 ppm ozone were significantly greater than those with 0.24 ppm ozone bubbling for 1 min.

**Fig. 2:**
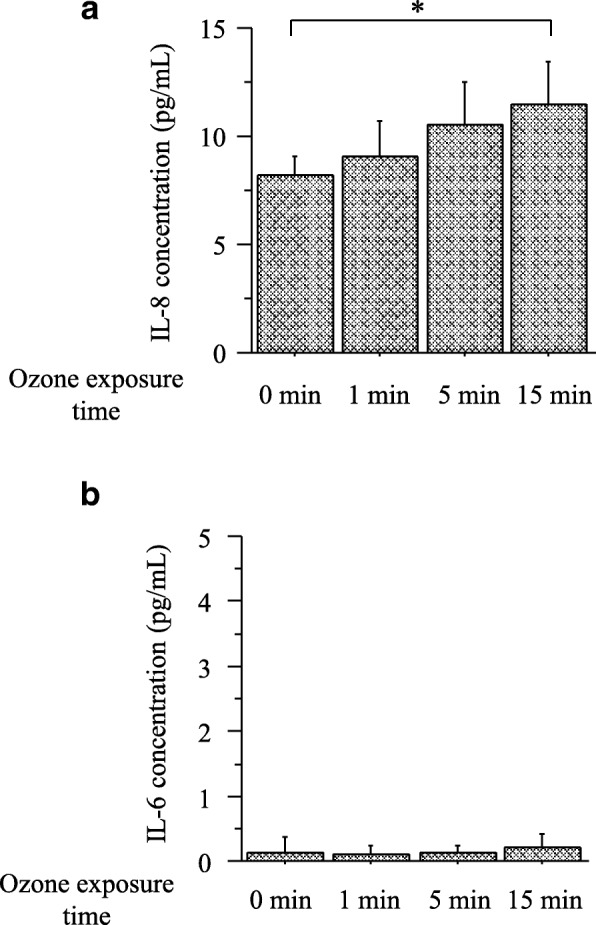
Simultaneous quantitative measurement of interleukin (IL)-8 (**a**) and IL-6 (**b**) induced by distilled deionized water with and without bubbling through 0.24 ppm ozone. Bars represent means ± SD. *P < 0.05

Figure 2 shows the simultaneous quantitative measurement of IL-6 and IL-8 induced by exposure to distilled deionized water with and without bubbling through 0.24 ppm ozone. The pH levels of distilled deionized water with and without bubbling through ozone ranged from 6.4 to 6.6. The concentrations of IL-8 were 8.2 ± 9.0 pg/mL without ozone exposure, 9.1 ± 1.7 pg/mL with ozone bubbling for 1 min, 10.6 ± 1.9 pg/mL for 5 min, and 11.5 ± 2.0 pg/mL for 15 min. There was a significant difference in the production of IL-8 between distilled deionized water without bubbling through ozone and that with 15 min of bubbling (*P* < 0.05). The concentration of IL-6 did not increase by distilled deionized water with and without bubbling through ozone.

**Fig. 3:**
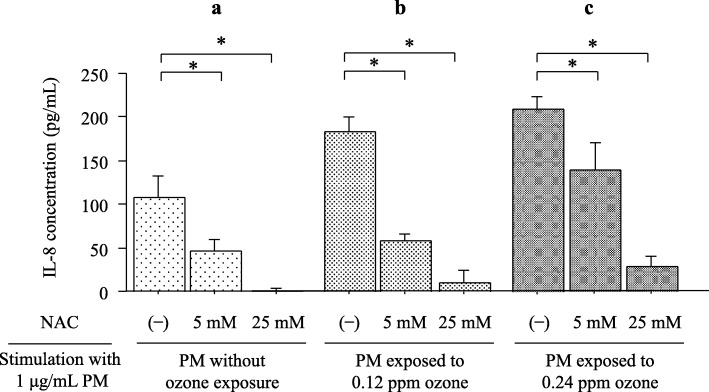
Effect of *N*-acetyl-_L_-cysteine (NAC) on the increase in interleukin (IL)-8 production by collected PM dissolved in solution (1 μg/mL) without bubbling through ozone (**a**) and with bubbling through 0.12 ppm ozone (**b**) and 0.24 ppm ozone (**c**). The antioxidant NAC significantly inhibited the production of IL-8 induced by all collected PM dissolved in solution in a dose-dependent manner. Bars represent means ± SD. * *P* < 0.01

Figure 3 summarizes the effect of NAC on the increase in IL-8 production by collected PM dissolved in solution with and without bubbling through ozone. The presence of the antioxidant NAC can significantly inhibit the increases in IL-8 induced by all collected PM dissolved in solution in a dose-dependent manner.

### Formation of ROS by distilled deionized water and PM dissolved in solution

**Fig. 4:**
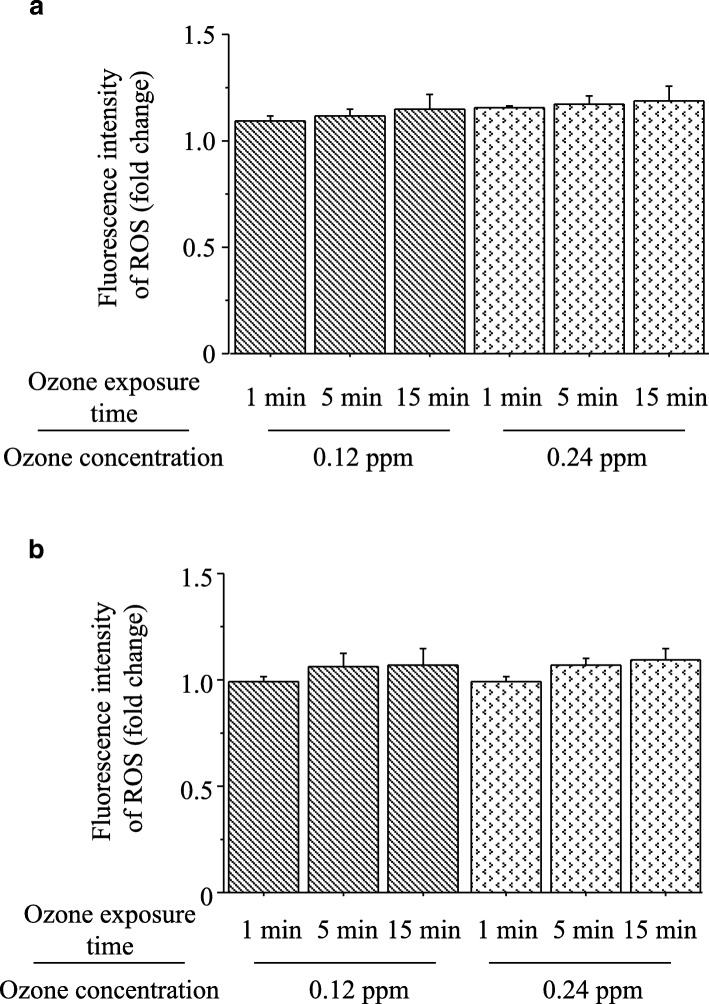
Formation of ROS by ozone exposure was assessed based on the fold induction of the fluorescein intensity using the 2′,7′-dichlorodihydrofluorescein diacetate reagent, which was calculated as the fluorescein intensities of distilled deionized water and PM dissolved in solution with bubbling through 0.12 ppm and 0.24 ppm ozone for 1 min, 5 min, and 15 min divided by those without bubbling through ozone. **a** Generation of ROS by ozone in collected PM dissolved in solution. **b** Generation of ROS by ozone in distilled deionized water. Bars represent means ± SD

The ability of ozone to generate ROS in distilled deionized water and PM dissolved in solution was assessed using fluorescein reagents. The generation of ROS by ozone exposure is shown in Fig. 4. The formation of ROS was assessed based on the fold induction of fluorescein intensity, which was calculated as the fluorescein intensity of distilled deionized water and PM dissolved in solution with bubbling through ozone divided by that without bubbling through ozone, respectively [42]. Ozone did not generate ROS using either distilled deionized water and PM dissolved in solution.

## Discussion

Ambient PM has the potential to produce pro-inflammatory cytokines and its toxicological mechanisms are important for understanding its adverse effects on human health. According to experimental and epidemiological studies, there is a combined effect of fine PM and ozone on health. However, the influence of ozone on the effects of PM on pro-inflammatory cytokine production is unclear. The effect of ozone on the production of IL-6 and IL-8 by collected ambient PM smaller than 1 μm in diameter was assessed after bubbling through ozone. Our key finding was that ozone significantly augmented the production of IL-8 by PM depending on the concentration of ozone. In contrast, ozone did not influence IL-8 production using distilled deionized water, a solvent for collected PM dissolved in solution. These results suggested that ozone may be able to change the chemical behavior of ambient PM and augment the production of pro-inflammatory cytokines in response to ambient PM.

Numerous experimental studies have demonstrated that ambient PM can induce IL-6, but IL-6 production was not detected in our study. Our previous study showed that the production of IL-6 in response to ambient PM in THP1 cells was lower than the production of IL-8 [43]. The lack of IL-6 production in the present study may be explained by the low concentration of ambient PM, i.e., 1 μg/mL; most studies have used more than 10 μg/mL PM to investigate the production of pro-inflammatory cytokines in response to ambient PM [12–15, 22–26]. Airborne PM is usually categorized based on particle size and is classified as primary and secondary PM [44]. Secondary PM is smaller than primary PM and there seems to be a division at an aerodynamic diameter of about 1 μm between primary and secondary PM [44]. The present study focused on the combined effects of PM smaller than 1 μm in diameter and ozone on pro-inflammatory cytokine production, but the quantity of ambient PM collected was insufficient.

In general, ambient PM promotes ROS production and activates ROS-generating pathways in cells [45]. ROS-generating pathways can lead to the activation of mitogen-activated protein kinases (MAPKs) and central pro-inflammatory transcription factors, such as nuclear factor-kappa B (NF-κB) and activator protein 1 (AP-1), leading to the up-regulation of pro-inflammatory genes, including various cytokines and chemokines. ROS is generated directly by reactive PM surfaces [13]. The level of ROS in the epithelial lining fluid covering the airways increases and is sustained by exposure to ambient PM [46, 47]. Therefore, in the present study, associations with IL-8 production and ROS were evaluated to investigate the mechanisms by which ozone augmented IL-8 production in response to ambient PM. In the present study, the antioxidant NAC significantly inhibited the increases in IL-8 induced by ambient PM, but exposure to ozone was not associated with the generation of ROS on PM. Thus, we should consider mechanisms underlying the augmentation of IL-8 production in response to ambient PM after exposure to ozone other than the generation of ROS on PM. Ambient PM is composed of a complex mixture originating from multiple sources, and gaseous air pollutants, such as ammonium (NH_3_), volatile organic compounds, sulfur oxides, and nitrogen oxides, chemically react with each other to generate secondary ambient PM [48–50]. Ozone may change the chemical compositions of PM and its augmented ROS-generating pathways. It may be useful to compare the chemical compositions of PM between before and after exposure to ozone to determine the underlying mechanisms. However, we were unable to analyze the chemical compositions of PM owing to an insufficient amount of PM; this was a limitation of the study.

Many experimental studies have used high concentrations of ozone, ranging from 0.2 ppm to 0.8 ppm, which greatly exceed environmental standards. In the present study, PM was exposed to ozone at Japanese warning and alarm levels, i.e., 0.12 ppm and 0.24 ppm, respectively. High levels of photochemical ozone have recently been decreasing in Japan, but an upward trend continues for the average annual level [51]. The area for the photochemical smog warning has increased in Japan. Our findings suggest that more attention should be paid to the potential adverse effects of ambient PM on health in these areas.

## Conclusions

In the present study, the influence of ozone at environmentally relevant concentrations on the production of pro-inflammatory cytokines in response to ambient PM were evaluated. Ozone significantly augmented the production of IL-8, but not IL-6, by THP1 cells in response to PM in a concentration-dependent manner. There may be a synergistic effect of ozone and ambient PM on inflammation.

## References

[1] Dockery DW, Pope CA, Xu X, Spengler JD, Ware JH, Fay ME (2003). An association between air pollution and mortality in six US Cities. N Engl J Med.

[2] Ware JH, Thibodeau LA, Speizer FE, Colome S, Ferris BG (1981). Assessment of the health effects of atmospheric Sulphur oxides and particulate matter: evidence from observational studies. Environ Health Perspect.

[3] Ghio AJ (2004). Biological effects of Utah Valley ambient air particles in humans: a review. J Aerosol Med.

[4] Atkinson RW, Kang S, Anderson HR, Mills IC, Walton HA (2014). Epidemiological time series studies of PM2.5 and daily mortality and hospital admissions: a systematic review and meta-analysis. Thorax.

[5] Weinmayr G, Romeo E, De Sario M, Weiland SK, Forastiere F (2010). Short-term effects of PM_10_ and NO_2_ on respiratory health among children with asthma or asthma-like symptoms: a systematic review and meta-analysis. Environ Health Perspect.

[6] Hong YC, Pan XC, Kim SY, Park K, Park EJ, Jin X (2010). Asian dust storm and pulmonary function of school children in Seoul. Sci Total Environ.

[7] Orellano P, Quaranta N, Reynoso J, Balbi B, Vasquez J. Association of outdoor air pollution with the prevalence of asthma in children of Latin America and the Caribbean: a systematic review and meta-analysis. J Asthma 2017. doi:10.1080/02770903.2017.1402342. [Epub ahead of print]. 10.1080/02770903.2017.1402342 29211546

[8] Atkinson RW, Butland BK, Dimitroulopoulou C, Heal MR, Stedman JR, Carslaw N (2016). Long-term exposure to ambient ozone and mortality: a quantitative systematic review and meta-analysis of evidence from cohort studies. BMJ Open.

[9] Kurt OK, Zhang J, Pinkerton KE (2016). Pulmonary health effects of air pollution. Curr Opin Pulm Med.

[10] Bell ML, Zanobetti A, Dominici F (2014). Who is more affected by ozone pollution? A systematic review and meta-analysis. Am J Epidemiol.

[11] Kelly FJ, Fussell JC (2011). Air pollution and airway disease. Clin Exp Allergy.

[12] Urch B, Speck M, Corey P, Wasserstein D, Manno M, Lukic KZ (2010). Concentrated ambient fine particles and not ozone induce a systemic interleukin-6 response in humans. Inhal Toxicol.

[13] Øvrevik J, Refsnes M, Låg M, Holme JA, Schwarze PE (2015). Activation of pro-inflammatory responses in cells of the airway mucosa by particulate matter: oxidant- and non-oxidant-mediated triggering mechanisms. Biomol Ther.

[14] Holgate ST, Sandström T, Frew AJ, Stenfors N, Nördenhall C, Salvi S (2003). Health effects of acute exposure to air pollution. Part I: healthy and asthmatic subjects exposed to diesel exhaust. Res Rep Health Eff Inst.

[15] Kafoury RM, Kelley J (2005). Ozone enhances diesel exhaust particles (DEP)-induced interleukin-8 (IL-8) gene expression in human airway epithelial cells through activation of nuclear factors- kappaB (NF-kappaB) and IL-6 (NF-IL6). Int J Environ Res Public Health.

[16] Devlin RB, McKinnon KP, Noah T, Becker S, Koren HS (1994). Ozone-induced release of cytokines and fibronectin production by alveolar macrophages and airway epithelial cells. Am J Phys.

[17] Balmes JR, Chen LL, Scannell C, Tager I, Christian D, Hearne PQ (1996). Ozone-induced decrements in FEV1 and FVC do not correlate with measures of inflammation. Am J Respir Crit Care Med.

[18] Seltzer J, Bigby BG, Stulbarg M, Holtzman MJ, Nadel JA, Ueki IF (1986). O3-induced change in bronchial reactivity to methacholine and airway inflammation in humans. J Appl Physiol.

[19] Kafoury RM, Pryor WA, Squadrito GL, Salgo MG, Zou X, Friedman M (1998). Lipid ozonation products activate phospholipases A2, C, and D. Toxicol Appl Pharmacol.

[20] Kafoury RM, Pryor WA, Squadrito GL, Salgo MG, Zou X, Friedman M (1999). Induction of inflammatory mediators in human airway epithelial cells by lipid ozonation products. Am J Respir Crit Care Med.

[21] Pryor WA, Squadrito GL, Friedman M (1995). The cascade mechanism to explain ozone toxicity: the role of lipid ozonation products. Free Rad Biol Med.

[22] Vincent R, Bjarnason SG, Adamson IY, Hedgecock C, Kumarathasan P, Guenette J (1997). Acute pulmonary toxicity of urban particulate matter and ozone. Am J Pathol.

[23] Bouthillier L, Vincent R, Goegan P, Adamson IY, Bjarnason S, Stewart M, et al. Acute effects of inhaled urban particles and ozone: lung morphology, macrophage activity, and plasma endothelin-1. Am J Pathol. 153:1873–84. 10.1016/S0002-9440(10)65701-X PMC1866316 9846977

[24] Adamson IY, Vincent R, Bjarnason SG (1999). Cell injury and interstitial inflammation in rat lung after inhalation of ozone and urban particulates. Am J Respir Cell Mol Biol.

[25] Wang G, Zhao J, Jiang R, Song W (2015). Rat lung response to ozone and fine particulate matter (PM_2.5_) exposures. Environ Toxicol.

[26] Auten RL, Potts EN, Mason SN, Fischer B, Huang Y, Foster WM (2009). Maternal exposure to particulate matter increases postnatal ozone-induced airway hyperreactivity in juvenile mice. Am J Respir Crit Care Med.

[27] Bosson J, Barath S, Pourazar J, Behndig AF, Sandstrom T, Blomberg A, Adelroth E (2008). Diesel exhaust exposure enhances the ozone-induced airway inflammation in healthy humans. Eur Respir J.

[28] Brook RD, Brook JR, Urch B, Vincent R, Rajagopalan S, Silverman F (2002). Inhalation of fine particulate air pollution and ozone causes acute arterial vasoconstriction in healthy adults. Circulation.

[29] Urch B, Brook JR, Wasserstein D, Brook RD, Rajagopalan S, Corey P, Silverman F (2004). Relative contributions of PM_2.5_ chemical constituents to acute arterial vasoconstriction in humans. Inhal Toxicol.

[30] Urch B, Silverman F, Corey P, Brook JR, Lukic KZ, Rajagopalan S, Brook RD (2005). Acute blood pressure responses in healthy adults during controlled air pollution exposures. Environ Health Perspect.

[31] Bell ML, Kim JY, Dominici F (2007). Potential confounding of particulate matter on the short-term association between ozone and mortality in multisite time-series studies. Environ Health Perspect.

[32] Franklin M, Schwartz J (2008). The impact of secondary particles on the association between ambient ozone and mortality. Environ Health Perspect.

[33] Anderson GB, Krall JR, Peng RD, Bell ML (2012). Is the relation between ozone and mortality confounded by chemical components of particulate matter? Analysis of 7 components in 57 US communities. Am J Epidemiol.

[34] Zhou J, Ito K, Lall R (2011). Time-series analysis of mortality effects of fine particulate matter components in Detroit and Seattle. Environ Health Perspect.

[35] Schlesinger RB (2007). The health impact of common inorganic components of fine particulate matter (PM_2.5_) in ambient air: a critical review. Inhal Toxicol.

[36] Valavanidis A, Fiotakis K, Vlachogianni T (2008). Airborne particulate matter and human health: toxicological assessment and importance of size and composition of particles for oxidative damage and carcinogenic mechanisms. J Environ Sci Health C Environ Carcinog Ecotoxicol Rev.

[37] Zhang Q, Jimenez JL, Canagaratna MR, Allan JD, Coe H, Ulbrich I (2007). Ubiquity and dominance of oxygenated species in organic aerosols in anthropogenically-influenced northern hemisphere midlatitudes. Gephysical Research Letters.

[38] Meng Z, Dabdub D, Seinfeld JH (1997). Chemical coupling between atmospheric ozone and particulate matter. Science.

[39] Hasei T, Watanabe T, Hirayama T (2006). Determination of 3,6-dinitrobenzo[e]pyrene in surface soil and airborne particles by high-performance liquid chromatography with fluorescence detection. J Chromatogr A.

[40] Iwata K, Watanabe M, Kurai J, Burioka N, Nakamoto S, Hantan D, Shimizu E (2017). Association between transported Asian dust and outdoor fungal concentration during winter in a rural area of western Japan. Genes Environ.

[41] Mitschik S, Schierl R, Nowak D, Jörres RA (2008). Effects of particulate matter on cytokine production in vitro: a comparative analysis of published studies. Inhal Toxicol.

[42] Onuma K, Sato Y, Ogawara S, Shirasawa N, Kobayashi M, Yoshitake J (2009). Nano-scaled particles of titanium dioxide convert benign mouse fibrosarcoma cells into aggressive tumor cells. Am J Pathol.

[43] Watanabe M, Noma H, Kurai J, Sano H, Hantan D, Ueki M (2016). Effects of short-term exposure to particulate air pollutants on the inflammatory response and respiratory symptoms: a panel study in schoolchildren from rural areas of Japan. Int J Environ Res Public Health.

[44] Whitby KT (1978). The physical characteristics of sulfer aerosols. Atomos Environ.

[45] David B, Armon RH, Hänninen O (2015). Environmental health: on the relationship between health outcome and urban air quality. Environmental Indicators.

[46] Gurgueira SA, Lawrence J, Coull B, Murthy GG, González-Flecha B (2002). Rapid increases in the steady-state concentration of reactive oxygen species in the lungs and heart after particulate air pollution inhalation. Environ Health Perspect.

[47] Lakey PS, Berkemeier T, Tong H, Arangio AM, Lucas K, Pöschl U, Shiraiwa M (2016). Chemical exposure-response relationship between air pollutants and reactive oxygen species in the human respiratory tract. Sci Rep.

[48] Bauer SE, Tsigaridis K, Miller R (2016). Significant atmospheric aerosol pollution caused by world food cultivation. Proc Geophys Res Lett.

[49] Heald CL, Collett JL, Lee T, Benedict KB, Schwandner FM, Li Y (2012). Atmospheric ammonia and particulate inorganic nitrogen over the United States. Atmos Chem Phys.

[50] Li L, Kumar M, Zhu C, Zhong J, Francisco JS, Zeng XC (2016). Near-barrierless ammonium bisulfate formation via a loop-structure promoted proton-transfer mechanism on the surface of water. J Amer Chem Soc.

[51] Japanese Meteorological Agency. Long–term trend of ozone. http://www.data.jma.go.jp/gmd/env/ozonehp/en/ltt_ozone.html. Accessed on 17 Apr 2018.

